# Assessing imputation techniques for missing data in small and multicollinear datasets: insights from craniofacial morphometry

**DOI:** 10.1186/s12874-025-02762-4

**Published:** 2026-02-04

**Authors:** Norli Anida Abdullah, Firdaus Hariri, Mohamad Norikmal Fazli Hisam, Siti Fatimah Binti Hassan

**Affiliations:** 1 Mathematics Division, Centre for Foundation Studies in Science, Universiti Malaya, Kuala Lumpur, Malaysia; 2 Center for Data Analytics Consultancy and Services, Faculty of Science, Universiti Malaya, Kuala Lumpur, Malaysia; 3 Department of Oral and Maxillofacial Clinical Sciences, Faculty of Dentistry, Universiti Malaya, Kuala Lumpur, Malaysia; 4 Institute of Mathematical Sciences, Faculty of Science, Universiti Malaya, Kuala Lumpur, Malaysia

**Keywords:** Craniofacial morphometry, Data imputation, Missing data, Multicollinear data, Variance preservation

## Abstract

**Background:**

Analyses of craniofacial morphology are essential for various medical and research applications, including the study of midfacial development, dysmorphologies, and planning surgical interventions. Incomplete CT scans often due to patient movement, imaging artifacts, or obscured landmarks which can result in missing data. If not properly addressed, such missingness may bias conclusions and weaken statistical power.

**Objective:**

This paper evaluates imputation techniques to identify the most suitable method for handling missing completely at random values in small, high-dimensional, and highly correlated craniofacial morphometric datasets.

**Methods:**

42 craniofacial variables were measured from 32 observations. The missing data structure was set to be at random with 268 (20%) missing values. Five common imputation techniques namely Mean/Median imputation, k-Nearest Neighbors (kNN), Multiple Imputation by Chained Equations (MICE), Random Forest (RF), and Decision Tree, were considered. The performance of the imputation technique was quantified using Root Mean Squared Error (RMSE), Mean Absolute Error (MAE), and Variance Preservation.

**Results:**

RF Imputation demonstrated the best overall performance, with the lowest RMSE (1.3987) and MAE (0.4902), indicating a high level of accuracy in imputing missing values. It also maintained a relatively close to 1 variance preservation (0.8961), suggesting its effectiveness in retaining the original variability in the dataset. MICE present lower accuracy with high RMSE (3.0869) and MAE (1.1246) however appear to have the closest variance preservation to 1 (1.0580).

**Conclusion:**

The findings emphasize the importance of choosing suitable imputation techniques for small, high-dimensional, and correlated datasets such as those in craniofacial morphometry. RF emerged as the most effective method, offering a strong balance between accuracy and variance preservation.

## Background

Analyses of craniofacial morphology are crucial for various medical and research applications, including the study of craniofacial development, dysmorphologies, and planning surgical interventions [1]. In the early stages of morphological research, animal models, particularly animal skulls, were extensively used to study craniofacial structures [2–4]. However, recent advancements have leveraged human skull datasets, providing more relevant and accurate insights for clinical and developmental studies [5, 6]. These datasets have become invaluable in various applications, ranging from surgical planning to the study of craniofacial anomalies.

Recent studies have developed statistical models to predict human skull growth using simple regression method [5], finite element method [7, 8], advanced machine learning or deep learning techniques [9, 10]. These advancements highlight the importance of comprehensive craniofacial datasets. Full sets of data are crucial for analysis and modelling to avoid biases and misinterpretations [11]. Accurate data is fundamental to making a reliable prediction and ensuring the validity of research outcomes [12]. Clavel, et al. [4] highlighted that even minor missing data in landmark-based analyses could result in significant errors in shape estimation and classification.

Acquiring complete and high-quality CT scans, along with precise measurements, can be challenging due to various factors that may arise during the scanning procedure or the measurement process. An incomplete CT scan can be due to the patient’s movement, existence of foreign objects during scanning procedure, or the limitations in imaging technology. During the measurement process, certain landmarks may be obscured or not visible in the scan, leading to missing measurements [13]. In practice, such missingness may not always be completely random, as certain regions, such as those near the edge of the scanner’s field of view, might be more prone to obscuration. In this study, however, we focused on a simulated Missing Completely at Random (MCAR) mechanism to represent cases where missingness could occur completely at random, such as from patient movement or hardware malfunction [13].

One of the most common strategies in clinical research to address missing data is Complete Case Analysis (CCA), where the case with missing values will be deleted and only cases with complete datasets are included in the analysis [14]. While CCA is simple and widely used, it can lead to substantial data loss and biased estimates [15]. In contrast, imputation methods such as Multiple Imputation by Chained Equations (MICE) and machine learning techniques like k-Nearest Neighbors (kNN) and Random Forest (RF), have been developed to estimate and replace missing values, thereby allowing the retention of incomplete cases in the analysis [16, 17]. While these techniques are commonly applied in various clinical and computational studies, their suitability for craniofacial morphometrics with small sample size and highly correlated variables remains an open question that this study seeks to address.

In the context of craniofacial research, particularly concerning the midface, addressing missing data is crucial due to the region’s complex anatomy and its significance in both functional and aesthetic aspects. The midface comprises structures such as the maxilla, zygomatic bones, and nasal cavity, which are integral to facial symmetry and function. Accurate morphometric analysis of this area is essential for understanding developmental anomalies, planning reconstructive surgeries, and conducting anthropological studies [18].

Given the critical role of craniofacial growth in both function and aesthetics, accurate morphometric analyses require robust methodologies that can handle missing data effectively. Functionally, craniofacial growth supports natural skull expansion and orbital wall development to accommodate brain growth and the eye globes. Additionally, the midfacial bones, maxilla, and mandible are closely related to upper airway patency and masticatory function, while their skeletal relationships also contribute substantially to overall facial appearance. Craniofacial morphometric data are typically characterized by a high number of interdependent variables (high dimensionality) relative to the number of observations (small sample size), resulting in pronounced multicollinearity. Studies show that different data structures present different challenges for imputation [19], as standard methods may be prone to overfitting, variance underestimation, or failure to preserve the underlying correlation structure [20]. Without proper handling, missing data can significantly distort the results of craniofacial studies and affect further development of analysis [21]. Imputation methods can effectively restore completeness in craniofacial CT datasets, reducing the impact of missing measurements caused by technical or clinical limitations, and providing clinicians with practical guidance for method selection, particularly valuable in rare conditions like syndromic craniosynostosis where data are often limited. This paper evaluates imputation techniques to identify the most suitable method for handling missing completely at random values in small, high-dimensional, and highly correlated craniofacial morphometric datasets. We review existing methodologies for handling missing data and evaluate their applicability to craniofacial morphometrics.

## Methodology

### Study database

#### Research ethics committee approval

This study was approved by the Medical Research Ethics Committee, University Malaya Medical Center (2022916-11546).

#### Sample selection

Selected computed tomography (CT) scan data were retrieved from a database of individuals who visited the University Malaya Medical Centre’s Craniofacial Clinic between November 2021 and December 2024. The inclusion criteria were pediatric subjects under 16 years of age with no history of surgical intervention, and a complete facial structure as captured on CT scans. A total of 32 patients, aged between 4 and 190 months (mean age 64.03 ± 58.14 months), were included in the study.

The CT scans were then uploaded to Materialize Mimics Medical software version 21.0 (Materialize, Leuven, Belgium) which converted the CT scans into 3D skull models. Anatomical landmarks were identified on the models, and the software automatically calculated the relevant angular and linear measurements between these landmarks. A total of 42 different linear and angular measurements were collected. The measurements are categorized in Table 1, and the landmarks are fully explained in Appendix (1) In addition, a figure showing the skull surface images with the landmarks is included in Appendix (2) The dataset can be accessed at the following link: 10.22452/RD/4YR5BX.

**Table 1:** Details on craniofacial measurements selected from 32 normal patients

Region	Variables	Notes
Nasal	ALL-ALR, N-Ro, Ro-ANS	Focused on nasal ridge, tip, and base
Maxilla	ANS-CP, ANS-SMP, ANS-SP, JL-JR, TM-CP, TM-SP, TM-MO, TMR-CP, TMR-SP, TM-TMR, TMR-MOR	ANS- and TM-related distances, often to midline or coronal planes
Orbital Floor	MO-CP, MO-MOR, MO-SP, MOR-CP, MOR-SP	Maxilla-to-orbit distances, orbital floor-to-suture relations
Zygomatic	ZMs-CP, ZMs-SP, ZMs-ZMsR, ZMs-MO, ZMsR-CP, ZMsR-SP, ZMsR-MOR	Zygomatic ridge and orbital rim contacts
Maxillary Transition	ZMi-CP, ZMi-SP, ZMi-ZMiR, ZMi-ZMs, ZMiR-CP, ZMiR-SP, ZMiR-ZMsR	Zygomatic-maxillary suture and transition-related measures
Midline/Frontal Suture	SMF-SMFR	Frontal suture measurement
Composite Angles	N-Ro-ANS, SNA, ZMs-MO-TM, ZMsR-MOR-TMR, MOR-TMR-PNS, MO-TM-PNS	Angular (3-point) measures; anchors in nasal, midline, zygomatic, and orbital regions

### Eligibility criteria

**Fig. 1:**
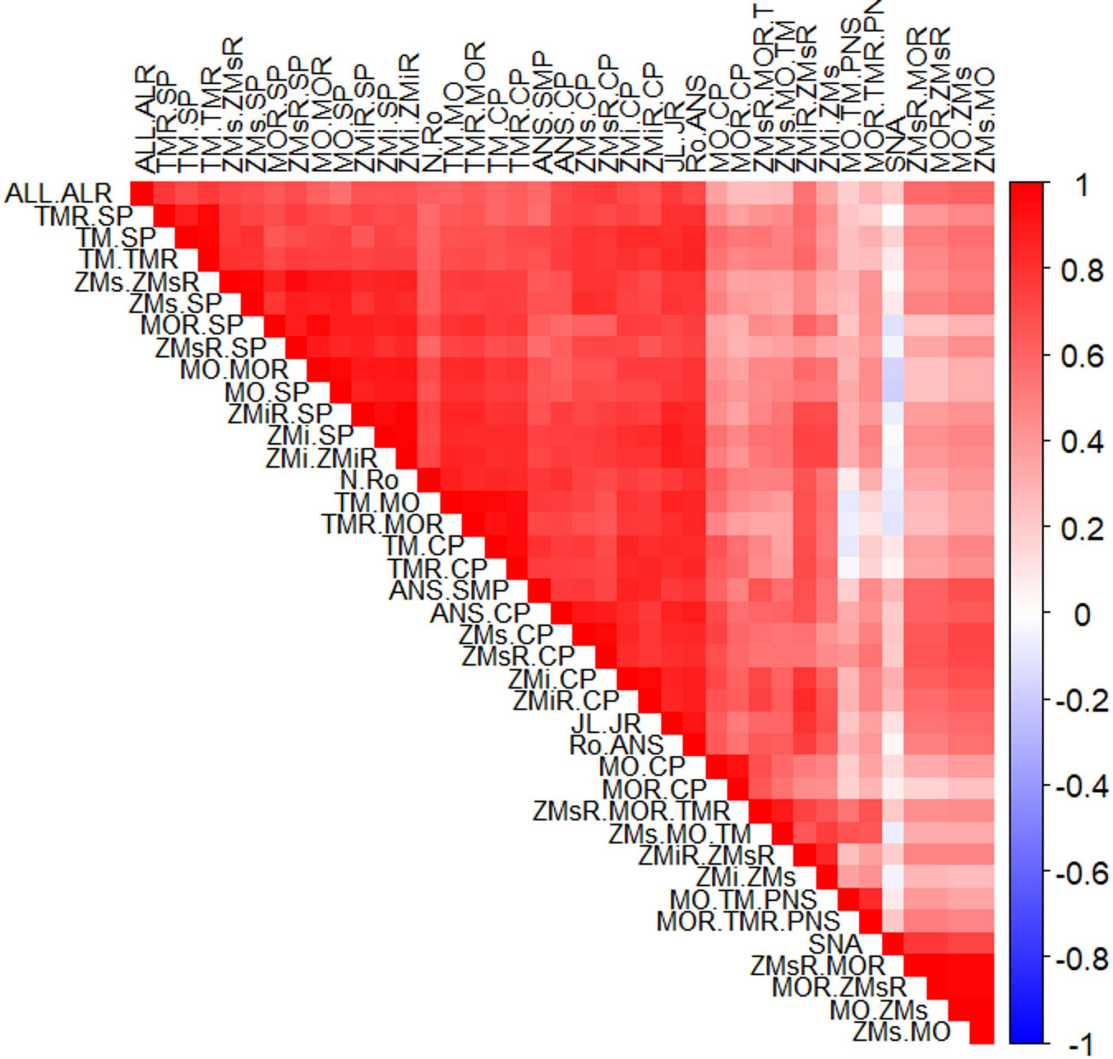
Correlation heatmap of the pairwise correlations above a threshold of |*r*| > 0.7

We assessed the correlation structure of the complete craniofacial dataset to understand interdependencies among variables. Given the anatomical proximity and symmetry of many craniofacial landmarks, substantial correlations were expected. A filtered correlation heatmap (Fig. 1) was generated to visualize pairwise correlations above a threshold of *|r| > 0.7*. The presence of highly correlated variable pairs supports the need for imputation methods able to handle multicollinearity effectively. The combination of a small sample size (*n* = 32) and high dimensionality (42 variables) further complicates the imputation process, as many variables are highly correlated. Thus the risk of overfitting is substantial.

### Missing data structure

**Fig. 2:**
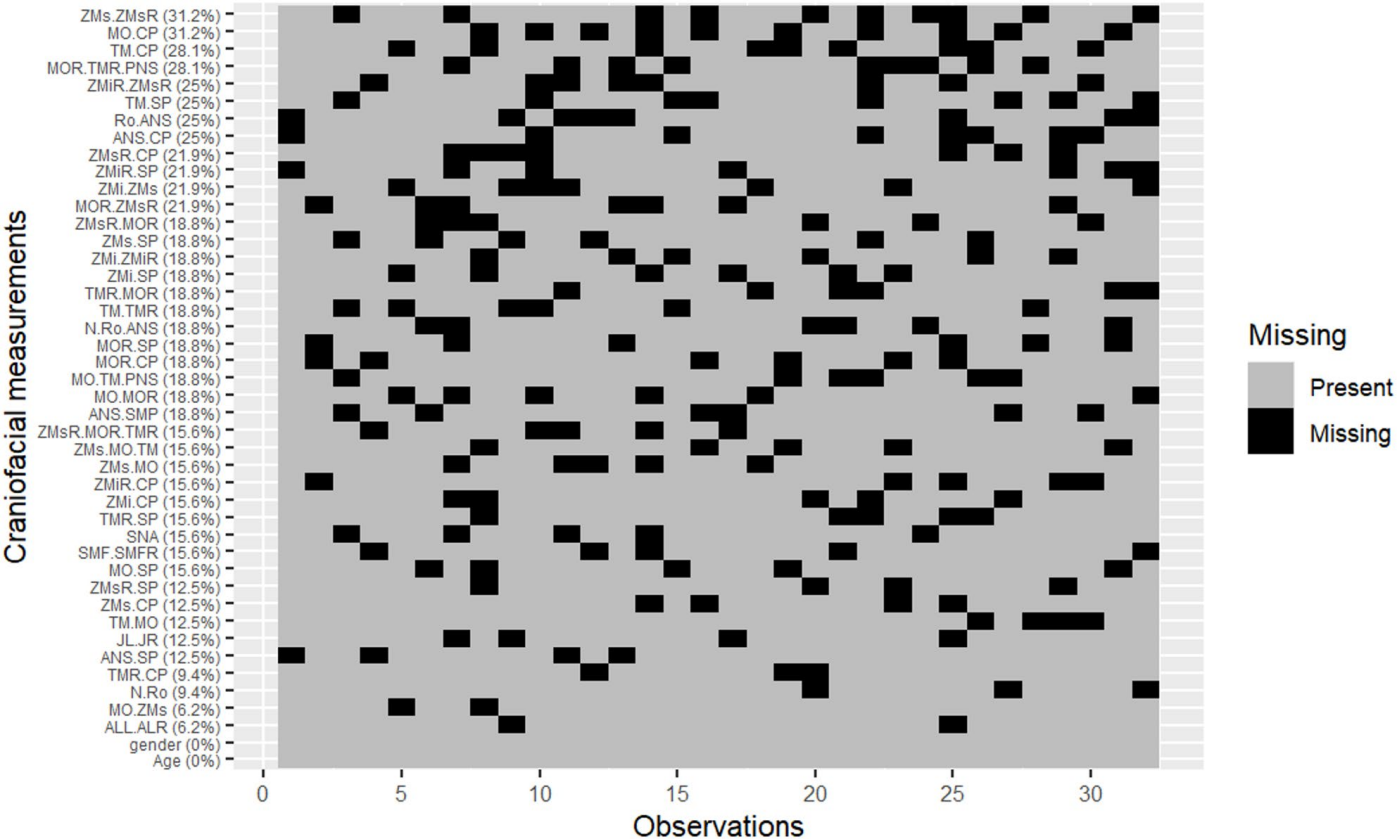
Heat map of 23 variables with missing data of 24 normal human skulls. A Missing Completely at Random (MCAR) structure with 20% missingness was introduced, showing that missing values are randomly distributed and independent of one another

In this study, we introduced missingness into the midface measurement dataset under the Missing Completely At Random (MCAR) assumption. To simulate this, we implemented a custom R function that randomly assigned missing values across selected variables using the sample() function. A total of 20% missingness was introduced by computing the number of values to be removed as a proportion of all eligible data cells and then randomly sampling their positions to replace with NA. This approach ensured a non-systematic pattern of missingness, consistent with the MCAR definition, and was performed using a fixed random seed (set.seed(123)) to maintain reproducibility. The choice of a 20% missingness level reflects a moderate degree of data loss commonly encountered in real-world clinical imaging datasets and is consistent with prior simulation-based evaluations of imputation methods [22]. Table 2 summarizes the data set of measurements with missing data for each variable while Fig. 2 shows the pattern of missingness. From Fig. 2, it is evident that the data are missing completely at random and independent of other missing values.

**Table 2:** Descriptive statistics for each variable after introducing missing completely at random (MCAR) structure to the craniofacial dataset

Region	Variables	Count	Percentage of missingness	Mean	Variance	Chi-Square (*P*-Values)
Composite Angles	MOR-TMR-PNS	9	28.13%	72.83	66.55	0.8246
	MO-TM-PNS	6	18.75%	70.68	48.02	0.1256
	N-Ro-ANS	6	18.75%	149.34	108.47	0.5326
	SNA	5	15.63%	71.26	85.24	0.4104
	ZMs-MO-TM	5	15.63%	65.35	84.06	0.8788
	ZMsR-CP	7	21.88%	21.35	13.2	1.0000
Maxilla	ANS-CP	8	25.00%	31.9	78.86	0.5395
	ANS-SMP	6	18.75%	27.65	16.32	0.7767
	ANS-SP	4	12.50%	0.86	0.69	0.1408
	JL-JR	4	12.50%	54.22	51.61	0.6879
	TM-CP	9	28.13%	30.58	49.08	0.3127
	TM-MO	4	12.50%	23.84	39.69	0.0818
	TMR-CP	3	9.38%	30.34	40.87	0.9093
	TMR-MOR	6	18.75%	22.98	23.63	0.1256
	TMR-SP	5	15.63%	21.14	10.75	0.2593
	TM-SP	8	25.00%	21.12	10.63	1.0000
	TM-TMR	6	18.75%	43.54	38.62	0.2336
Maxillary Transition	ZMi-CP	5	15.63%	28.68	34.73	0.8788
	ZMiR-CP	5	15.63%	28.51	25.71	0.2593
	ZMiR-SP	7	21.88%	33.87	17.12	1.0000
	ZMiR-ZMsR	8	25.00%	19.43	13.26	0.8379
	ZMi-SP	6	18.75%	33.81	27.05	0.7767
	ZMi-ZMiR	6	18.75%	67.78	105.29	1.0000
	ZMi-ZMs	7	21.88%	18.91	13.4	1.0000
Midline/Frontal Suture	SMF-SMFR	5	15.63%	15.24	4.29	1.0000
Nasal	ALL-ALR	2	6.25%	17.09	6.76	1.0000
	N-Ro	3	9.38%	14.96	17.6	0.1838
	Ro-ANS	8	25.00%	19.95	15.44	0.8379
Orbital Floor	MO-CP	10	31.25%	7.95	3.58	1.0000
	MO-MOR	6	18.75%	38.13	55.19	0.7767
	MOR-CP	6	18.75%	8.43	4.24	1.0000
	MOR-SP	6	18.75%	18.97	13.93	1.0000
	MOR-ZMsR	7	21.88%	20.75	30.19	0.1269
	MO-SP	5	15.63%	18.63	14.47	1.0000
	MO-ZMs	2	6.25%	21.65	29.26	0.5220
Zygomatic	ZMs-CP	4	12.50%	21.44	16.84	1.0000
	ZMs-MO	5	15.63%	21.62	31.63	0.4104
	ZMsR-MOR	6	18.75%	21.01	30.04	1.0000
	ZMsR-MOR-TMR	5	15.63%	67.32	93.86	0.0720
	ZMsR-SP	4	12.50%	22.27	10.88	0.5032
	ZMs-SP	6	18.75%	22.58	19.83	0.7767
	ZMs-ZMsR	10	31.25%	43.85	55.57	0.5346

**Fig. 3:**
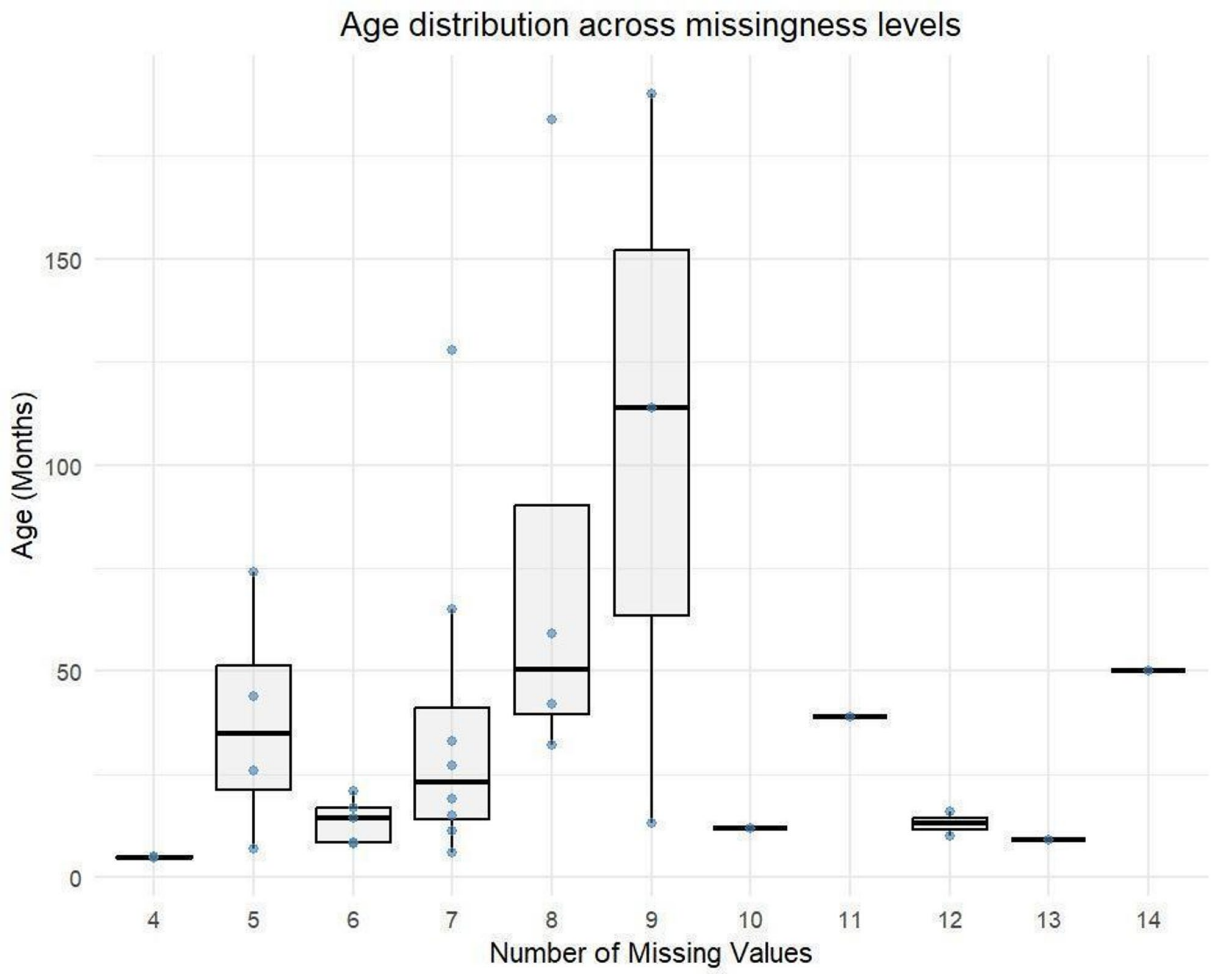
Age distribution across missingness levels. Variation in age shows no clear trend with missingness, supporting the assumption that the data are MCAR rather than MAR

To confirm the MCAR assumption, a chi-square test was performed. The p-values in Table 2 indicate insufficient evidence to reject the null hypothesis, supporting that the data are Missing Completely at Random (MCAR). This is further illustrated in Fig. 3, where the box-and-dot plot shows that age distribution does not vary across missingness levels, suggesting that missingness is completely random.

### Imputation methods of missing data

This subsection briefly details five common imputation methods for handling missing data estimates and replacing missing values based on existing data.

1. Simple imputation (mean/median): Missing data is assigned with a quantitative or qualitative value related to the non-missing part. It is common for continuous datasets to use mean or median to replace missing values and it is one of the simplest imputation methods. However, it can reduce data variability and introduce bias [23].
2. kNN imputation: Missing values are estimated using the nearest observed cases within the dataset. For each missing value, kNN identifies *k* closest neighbours using a distance matrix (such as Euclidean and Manhattan), then imputes the missing value using the mean of the nearest neighbours [24].
3. MICE: Model-based imputation that estimates missing values iteratively using a series of predictive models. MICE initializes missing values using a simple imputation; then the value is iteratively modelled as a function of all other variables using regression-based approaches and the imputed values are updated sequentially. MICE can use various algorithms to impute data, but the most common is multiple imputation using chained equations [25].
4. RF imputation: RF imputation is a tree-based machine learning approach that predicts missing values using a trained RF model. The method uses observed data to construct decision trees and predict missing values based on the patterns in the available data [26].
5. Decision Tree Imputation: Decision Tree imputation applies tree-based models to predict missing values by splitting data into homogeneous groups based on observed features [27]. It is non-parametric, meaning it does not assume a specific data distribution, making it useful for datasets with complex missingness patterns. Decision trees can handle both categorical and continuous variables and are robust to outliers.

### Performance evaluation of the imputation methods

It is crucial to select an imputation method that minimizes distortion, preserves variance, and maintains the statistical properties of the original dataset. To assess the effectiveness of each imputation method, multiple evaluation metrics were employed such as Root Mean Squared Error (RMSE), Mean Absolute Error (MAE), and variance preservation.

#### Root Mean Squared Error (RMSE)

RMSE measures the average squared difference between the original and estimated values, providing a robust measure of error magnitude. The RMSE is computed as follows:

1 $$RMSE=\:\sqrt{\frac1n\sum_{i=i}^n\left(y_i-{\widehat y}_i\right)^2}$$

#### Mean Absolute Error (MAE)

MAE calculates the absolute difference between the observed and estimate values using the formula:

2 $$\:MAE=\sqrt{\frac1n\sum_{i=1}^n\vert y_i-{\widehat y}_i\vert}\:$$

where $\:{y}_{i}$ is the observed value, $\:{\widehat{y}}_{i}$ denotes the imputed value, and $\:n$ is the total number of missing values.

Lower RMSE and MAE values indicate a closer approximation to the original data, thereby signifying a more reliable imputation method.

#### Variance preservation

While RMSE and MAE quantify the average error between imputed and true values, they do not reflect whether the spread or variability of the data is maintained. This can be misleading; a method may yield low RMSE but compresses the variability of key morphometric features, distorting the shape space. Variance preservation is quantified for each variable as the ratio of post-imputation to original variance. The closer the variance of the imputed dataset to the original measurement, the more accurate the method. Variance is calculated using:

3 $$\:Var\left(y\right)=\:\frac1{m-1}\sum_{i=i}^n({y_i-{\overline{y}}_i)}^2$$

where $\:{\overline{y}}_{i}$ is the mean of the observed value and $\:m$ is the number of observations. The difference of this variance with the original variance is compared. Small changes indicate high variance preservation. Therefore, variance preservation is given by:

4 $$\:variance\:preservation=\:\frac{Imputed\:Var\left(y\right)}{Original\:Var\left(y\right)}$$

## Results

### Performance evaluations of data imputation methods

Table 3 presents the mean and standard deviation of each variable before and after imputation using different methods, including Mean Imputation, Median Imputation, RF, kNN, Decision Tree, and MICE. From the table, we observe that all imputation methods successfully preserve the mean values of most variables with minor variations. However, differences in standard deviations indicate that some methods may have altered the data’s variability. Mean and Median Imputation often result in a slight reduction in standard deviation, suggesting a loss of variability due to their simplistic replacement strategies. RF and kNN Imputation appear to maintain variability more effectively, as their standard deviations are closer to the true values. MICE (Multiple Imputation by Chained Equations) appears to have the worst accuracy and the best variance preservation.

**Table 3:** Mean and standard deviations of imputed data before (True Value) and after imputation (Mean, Median, RF, kNN, decision tree and MICE)

Variable	True Value	Mean Imputation	Median Imputation	RF	kNN	Decision Tree	MICE
ALL-ALR	17.09 ± 2.6	17.09 ± 2.52	17.1 ± 2.52	17.12 ± 2.52	17.11 ± 2.52	17.05 ± 2.52	17.02 ± 2.53
ANS-CP	31.9 ± 8.88	31.9 ± 7.65	31.3 ± 7.72	32.92 ± 8.52	32.86 ± 8.01	32.45 ± 8.4	33.3 ± 9.62
ANS-SMP	27.65 ± 4.04	27.65 ± 3.63	27.49 ± 3.64	27.72 ± 3.82	27.66 ± 3.65	27.73 ± 3.88	27.08 ± 3.98
ANS-SP	0.86 ± 0.83	0.86 ± 0.77	0.8 ± 0.79	0.87 ± 0.78	0.8 ± 0.79	0.89 ± 0.78	1 ± 0.94
JL-JR	54.22 ± 7.18	54.22 ± 6.7	54.16 ± 6.71	54 ± 6.9	53.97 ± 6.79	53.98 ± 7.18	53.65 ± 7.73
MO-CP	7.95 ± 1.89	7.95 ± 1.56	7.85 ± 1.57	8.03 ± 1.69	8.03 ± 1.62	8.1 ± 1.7	7.75 ± 1.83
MO-MOR	38.13 ± 7.43	38.13 ± 6.67	38.29 ± 6.68	37.66 ± 7.25	37.34 ± 7.24	37.69 ± 7.13	38.01 ± 7.06
MOR-CP	8.43 ± 2.06	8.43 ± 1.85	8.45 ± 1.85	8.34 ± 1.91	8.32 ± 1.89	8.28 ± 2	8.32 ± 2.24
MOR-SP	18.97 ± 3.73	18.97 ± 3.35	18.87 ± 3.36	19.09 ± 3.7	18.83 ± 3.53	18.89 ± 3.59	18.91 ± 3.71
MOR-TMR-PNS	72.83 ± 8.16	72.83 ± 6.87	72.7 ± 6.88	73.37 ± 7.33	72.87 ± 7.11	73.25 ± 7.47	72.12 ± 9.13
MOR-ZMsR	20.75 ± 5.49	20.75 ± 4.83	20.43 ± 4.87	20.61 ± 5.19	21.01 ± 5.13	20.73 ± 5.38	20.75 ± 5.49
MO-SP	18.63 ± 3.8	18.63 ± 3.48	18.61 ± 3.48	18.67 ± 3.7	18.54 ± 3.67	18.36 ± 3.67	18.97 ± 4.33
MO-TM-PNS	70.68 ± 6.93	70.68 ± 6.22	70.52 ± 6.23	71.2 ± 6.5	71.08 ± 6.56	70.85 ± 6.4	70.18 ± 7.07
MO-ZMs	21.65 ± 5.41	21.65 ± 5.23	21.66 ± 5.23	21.35 ± 5.37	21.39 ± 5.33	21.37 ± 5.34	22.24 ± 5.72
N-Ro	14.96 ± 4.2	14.96 ± 3.99	14.93 ± 3.99	15.26 ± 4.25	15.06 ± 4.04	15.11 ± 4.1	14.78 ± 4.19
N-Ro-ANS	149.34 ± 10.41	149.34 ± 9.35	149.28 ± 9.35	148.98 ± 9.43	149.65 ± 9.51	149.51 ± 9.52	148.33 ± 11.51
Ro-ANS	19.95 ± 3.93	19.95 ± 3.38	19.91 ± 3.39	20.06 ± 4.02	19.88 ± 3.8	20.26 ± 3.73	20.03 ± 4.72
SMF-SMFR	15.24 ± 2.07	15.24 ± 1.9	15.22 ± 1.9	15.24 ± 1.94	15.24 ± 1.96	15.13 ± 2.05	15.04 ± 2.13
SNA	71.26 ± 9.23	71.26 ± 8.46	71.38 ± 8.46	71.4 ± 8.67	71.61 ± 8.72	71.08 ± 8.95	71.44 ± 9.52
TM-CP	30.58 ± 7.01	30.58 ± 5.9	29.87 ± 6.01	30.69 ± 6.21	30.11 ± 6.1	30.33 ± 6.37	30.21 ± 6.14
TM-MO	23.84 ± 6.3	23.84 ± 5.88	23.67 ± 5.9	24.7 ± 6.53	24.03 ± 5.96	24.56 ± 6.19	23.52 ± 6.06
TMR-CP	30.34 ± 6.39	30.34 ± 6.08	30.18 ± 6.1	30.22 ± 6.1	29.99 ± 6.19	30.57 ± 6.28	29.68 ± 6.48
TMR-MOR	22.98 ± 4.86	22.98 ± 4.37	22.78 ± 4.39	23.43 ± 4.77	23.17 ± 4.5	23.09 ± 4.68	23.55 ± 4.87
TMR-SP	21.14 ± 3.28	21.14 ± 3	21.06 ± 3.01	21.19 ± 3.05	21.18 ± 3.07	21.29 ± 3.24	21.57 ± 3.4
TM-SP	21.12 ± 3.26	21.12 ± 2.81	20.85 ± 2.85	21.39 ± 3.15	21.37 ± 3.07	21.3 ± 3.05	21.49 ± 3.3
TM-TMR	43.54 ± 6.21	43.54 ± 5.58	43.11 ± 5.65	43.08 ± 5.86	43.05 ± 5.86	43.21 ± 5.85	44.03 ± 6.81
ZMi-CP	28.68 ± 5.89	28.68 ± 5.4	28.5 ± 5.41	28.52 ± 5.46	28.77 ± 5.52	28.5 ± 5.59	28.86 ± 5.98
ZMiR-CP	28.51 ± 5.07	28.51 ± 4.64	28.26 ± 4.68	29 ± 5.12	28.63 ± 4.87	28.78 ± 4.94	28.66 ± 5.02
ZMiR-SP	33.87 ± 4.14	33.87 ± 3.64	34 ± 3.65	34.06 ± 4.27	33.91 ± 3.93	33.61 ± 4.02	33.75 ± 4.37
ZMiR-ZMsR	19.43 ± 3.64	19.43 ± 3.14	19.15 ± 3.17	19.33 ± 3.34	19.14 ± 3.25	19.36 ± 3.4	19.33 ± 3.7
ZMi-SP	33.81 ± 5.2	33.81 ± 4.67	33.71 ± 4.68	33.49 ± 4.8	33.42 ± 4.86	33.13 ± 4.89	33.9 ± 5.51
ZMi-ZMiR	67.78 ± 10.26	67.78 ± 9.21	67.67 ± 9.22	68.66 ± 9.66	68.13 ± 9.6	67.5 ± 9.86	68.34 ± 9.73
ZMi-ZMs	18.91 ± 3.66	18.91 ± 3.22	18.92 ± 3.22	18.74 ± 3.4	18.73 ± 3.38	18.88 ± 3.42	18.76 ± 3.9
ZMs-CP	21.44 ± 4.1	21.44 ± 3.83	21.38 ± 3.83	21.45 ± 3.86	21.32 ± 3.86	21.43 ± 4	20.85 ± 4.16
ZMs-MO	21.62 ± 5.62	21.62 ± 5.15	21.6 ± 5.15	21.27 ± 5.37	21.4 ± 5.24	21.5 ± 5.52	21.62 ± 5.62
ZMs-MO-TM	65.35 ± 9.17	65.35 ± 8.4	65.07 ± 8.42	65.75 ± 8.8	65.62 ± 8.83	65.15 ± 8.69	64.72 ± 10.07
ZMsR-CP	21.35 ± 3.63	21.35 ± 3.2	21.17 ± 3.22	21.54 ± 3.41	21.47 ± 3.38	21.36 ± 3.49	21.56 ± 3.88
ZMsR-MOR	21.01 ± 5.48	21.01 ± 4.92	20.81 ± 4.94	20.96 ± 5.13	21.08 ± 5.34	20.77 ± 5.37	21.02 ± 5.5
ZMsR-MOR-TMR	67.32 ± 9.69	67.32 ± 8.87	66.9 ± 8.93	66.62 ± 9.11	66.5 ± 9.1	67.13 ± 8.88	65.99 ± 10.81
ZMsR-SP	22.27 ± 3.3	22.27 ± 3.08	22.2 ± 3.08	22.44 ± 3.19	22.4 ± 3.14	22.22 ± 3.2	22.32 ± 3.1
ZMs-SP	22.58 ± 4.45	22.58 ± 4	22.49 ± 4	22.29 ± 4.16	22.47 ± 4.14	22.51 ± 4.27	23.33 ± 5.34
ZMs-ZMsR	43.85 ± 7.45	43.85 ± 6.14	43.48 ± 6.16	44.29 ± 6.73	43.73 ± 6.47	44.89 ± 7.12	44.52 ± 6.91

For specific variables, such as ANS-CP and MOR-TMR-PNS, the RF and kNN methods yield means and standard deviations closest to the original values, suggesting they might be more reliable for preserving the data structure. Conversely, Decision Tree and MICE show slight variations in certain cases, such as ZMs-ZMsR, while Decision Tree overestimates the standard deviation.

**Fig. 4:**
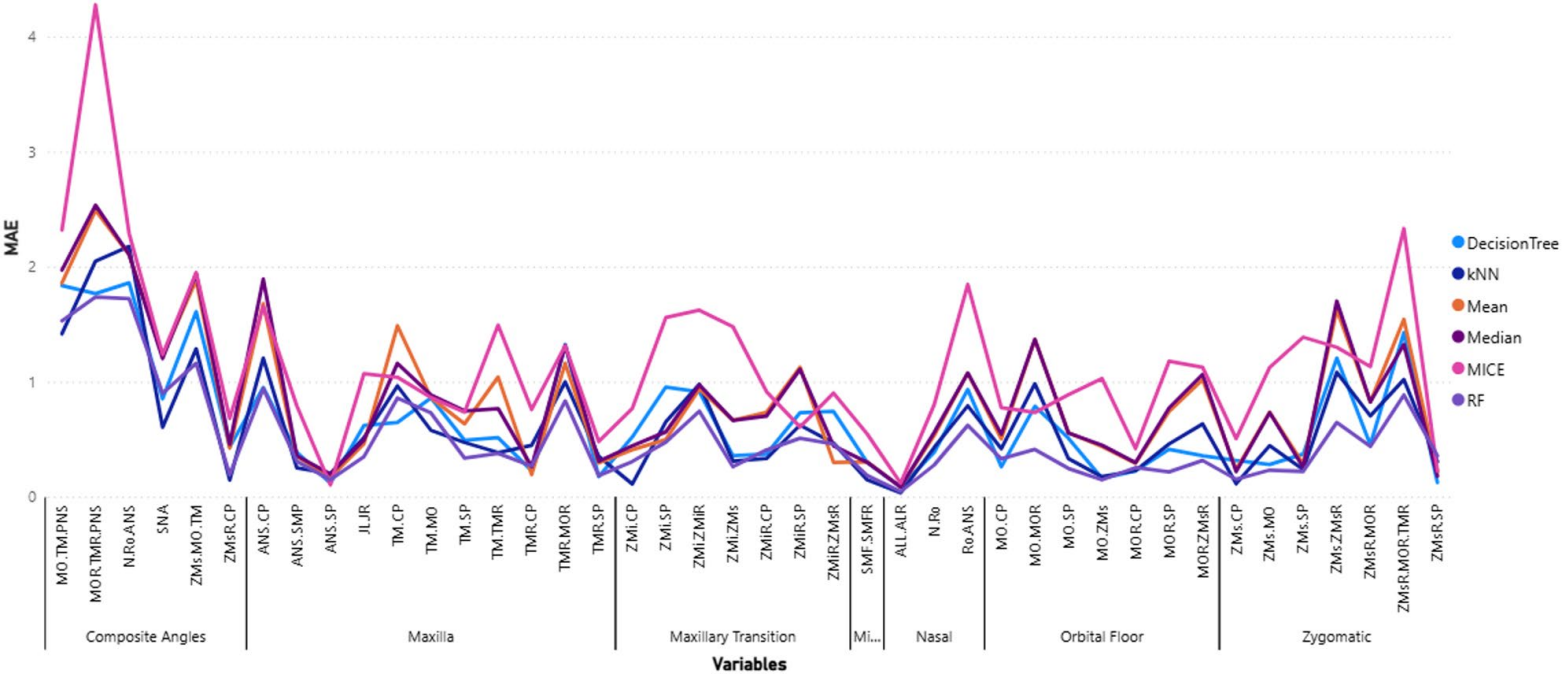
MAE for each imputed measurement across different regions

Figure 4 presents the MAE results for each craniofacial variable imputed using six different methods. The RF method outperformed all others, producing the lowest MAE in 24 out of 42 variables (57.14%), suggesting it offers the most accurate reconstructions relative to the original values. kNN was the second-best method, with the lowest MAE in 10 variables (23.81%), showing moderate effectiveness. In contrast, the Median imputation method did not yield the lowest MAE for any variable, indicating its limitations in handling the variability and complexity of craniofacial measurements. These findings highlight that machine learning–based methods, particularly RF, are more effective for this type of data than simpler statistical approaches.

**Fig. 5:**
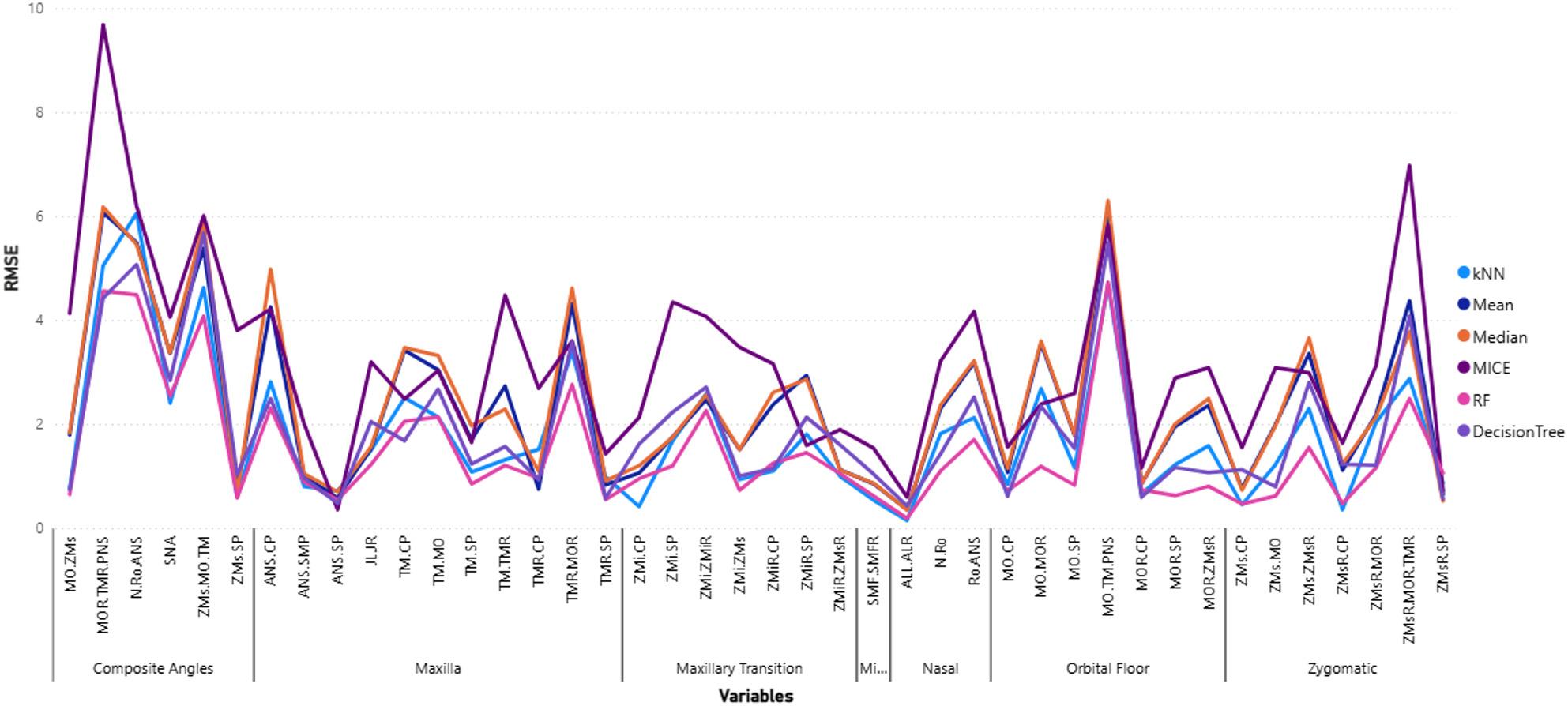
RMSE for each imputed measurement across different regions

Based on the RMSE results across 42 craniofacial variables in Fig. 5, RF imputation emerged as the most accurate method, achieving the lowest RMSE in 24 variables (57.14%). This was followed by kNN with 11 variables (26.19%), and Decision Tree in 4 variables (9.52%). RF’s superior performance can be attributed to its ability to model complex, non-linear relationships and variable interactions, making it well-suited for structured biomedical datasets. In contrast, deterministic methods like Mean and Median, and the iterative MICE approach, each only yielded the lowest RMSE in one variable (2.38%), indicating limited effectiveness in capturing the variability and multivariate dependencies inherent in craniofacial data.

**Fig. 6:**
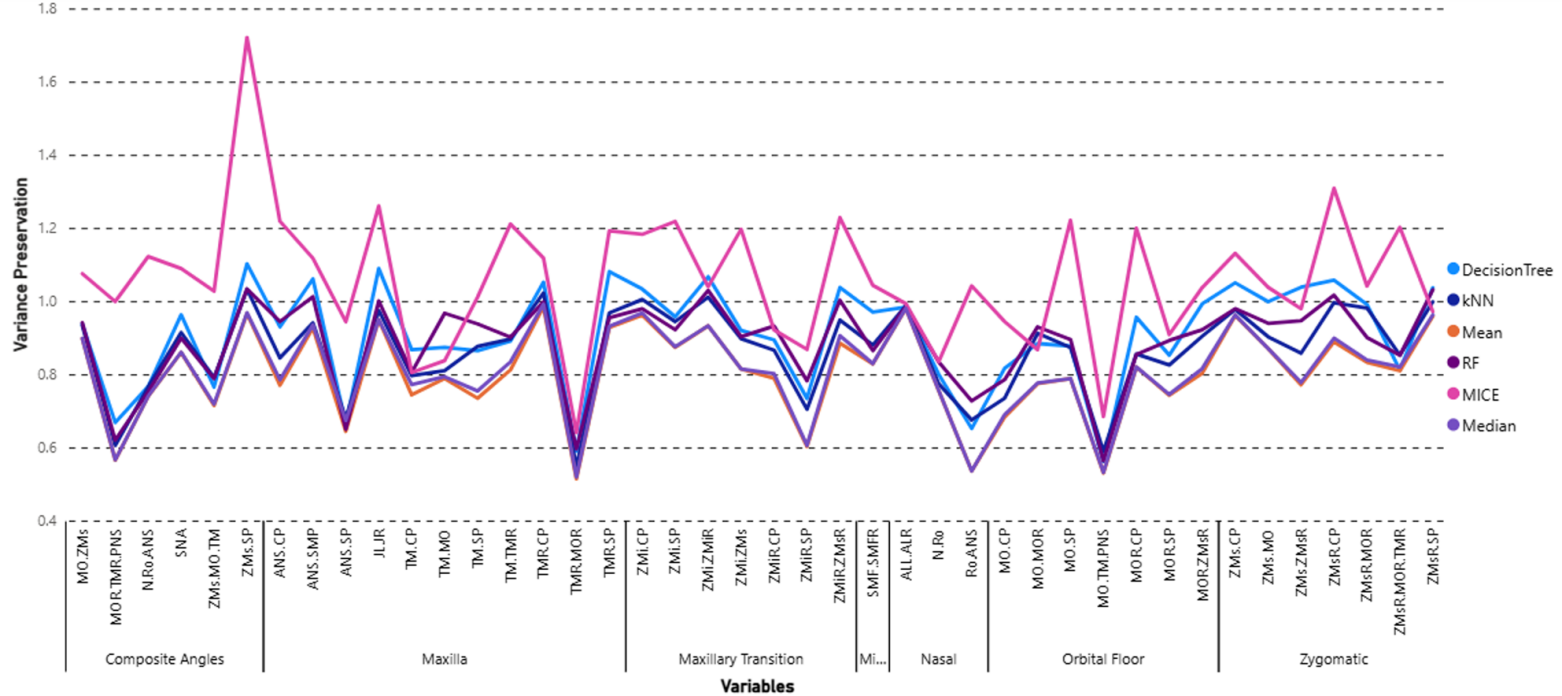
Variance preservation for each imputed measurement across different regions

While a method performed well based on RMSE and MAE, these metrics alone were misleading, as they did not account for the overall shape of the data distribution. In contrast, variance preservation revealed notable differences between imputation strategies. For example, some methods showed good average error (MAE/RMSE) but substantially reduced variance. Fig. 6 presents the variance preservation performance of each imputation method, where bolded values indicate the results closest to 1. Among the 42 craniofacial variables, MICE imputation achieved variance preservation values closest to 1 most often (14 out of 42 variables, 33.33%), followed by RF (28.57%), Decision Tree (21.43%), and kNN (14.29%). Mean imputation did not yield the best variance preservation for any variable, underscoring its limited ability to capture true variability in complex morphometric data.

Table 4 displays the RMSE, MAE and variance preservation score for various methods in imputation missing data. RF demonstrates the lowest RMSE (1.3987) and MAE (0.4902), indicating that it produces the most accurate imputations. However, its variance preservation (0.8961) is relatively far from 1. On the other hand, MICE presents lower accuracy with high RMSE (3.0869) and MAE (1.1246) with closest variance preservation to 1 (1.0580). This may potentially be due to overcorrection or instability in its imputation process.

**Table 4:** Mean of MAE, RMSE and variance preservation across different variables

Method	RMSE	MAE	Variance Preservation
Mean Imputation	2.3560	0.8418	0.8033
Median Imputation	2.4256	0.8522	0.8096
RF	1.4817	0.5170	0.8875
kNN	1.7925	0.6202	0.8656
Decision Tree	1.9140	0.6629	0.9082
MICE	3.1604	1.1514	1.0459

Our findings highlight the necessity of selecting imputation techniques suited to small-sample, high-dimensional, and highly multicollinear datasets, which are common in craniofacial morphometry. Consistent with previous research, machine learning-based approach such as RF demonstrated better overall accuracy and variance preservation compared to simpler methods, reflecting their ability to account for complex inter-variable dependencies. While MICE demonstrates notably worse imputation accuracy than all other methods, it provides the best variance preservation.

## Discussion

The findings of this study carry important clinical implications. In craniofacial practice, morphometric measurements derived from CT scans are frequently used to guide diagnosis, monitor growth, and plan surgical interventions. However, limited images, patient movement, or obscured landmarks often result in incomplete datasets, which may compromise clinical decision-making. Our results provide a foundation for understanding how different imputation methods behave under controlled conditions, which can offer clinicians guidance on method selection. The imputation restores dataset completeness and offers a practical tool to maximize the use of limited data, which is especially relevant in rare conditions such as syndromic craniosynostosis.

While methods like RF, Decision Tree, and MICE leverage inter-variable correlations to impute missing values, the high dimensionality and strong correlations typical of craniofacial datasets can introduce risks such as overfitting or unstable variance estimates, particularly when sample sizes are small. This study evaluates the performance of these imputation techniques in such a setting, highlighting their respective strengths and limitations in maintaining accuracy and preserving variance within craniofacial morphometric data. The Root Mean Squared Error (RMSE) and Mean Absolute Error (MAE) were used to assess imputation accuracy, while variance preservation was evaluated to determine how well each method retained the original data structure.

Among the methods tested, RF Imputation demonstrated the best overall performance, with the lowest RMSE (1.3987) and MAE (0.4902), indicating a high level of accuracy in reconstructing missing values. It also maintained a relatively high variance preservation score (0.8961), suggesting that it effectively retained the original variability in the dataset al.igning with previous studies that have shown RF to be an effective imputation technique, particularly in datasets with complex relationships and non-linearity [28]. Similarly, kNN and Decision Tree imputation performed reasonably well, with RMSE values of 1.7508 and 1.8695, respectively.

Decision Tree achieved the second closest variance preservation value to 1 (0.9082), indicating that it performs well in maintaining data variability when this is a key priority. Conversely, Mean and Median Imputation exhibited higher RMSE (2.3012 and 2.3692) and lower variance preservation (0.8079 and 0.8140), reflecting their inherent limitation of imputing missing values with constant values rather than leveraging relationships between variables. This agrees with prior research indicating that such simple imputation techniques tend to underestimate variance and introduce bias, especially in datasets with non-random missingness [29].

Interestingly, MICE resulted in variance preservation slightly exceeding 1 (1.0594) but still the closest to 1 compared to other methods, although it also showed the highest RMSE (3.1604) and MAE (1.1514). This suggests that while MICE maintains data variability well, it may slightly overestimate variance and introduce larger errors in individual imputations. This may be attributed to overfitting or sensitivity to the underlying imputation model, which has been observed in previous studies when using MICE in small or highly correlated datasets [30]. This is consistent with findings by Segura-Buisan, et al. [31] where multiple imputation methods such as MICE can effectively preserve variability but may produce less accurate estimates in complex or longitudinal settings with limited data.

These findings underscore the importance of effective missing-data handling in craniofacial morphometrics. With computational models increasingly used to predict craniofacial growth and deformities [5, 7, 9], reliable imputation is critical, particularly in clinical contexts where craniofacial measurements guide reconstructive surgery and orthodontic interventions [18]. Our results suggest that kNN or MICE imputation enhances dataset reliability and applicability, consistent with prior reports that inadequate handling of missing data can bias anatomical analyses [4].

This study was limited to MCAR missing data structures and craniofacial datasets, and the impact of imputation on clinical predictions was not assessed. Future work should extend analyses to other missing-data structures such as missing at random and missing not at random and evaluate their effect on clinical outcomes. Applying this framework to non-craniofacial datasets with similar characteristics (small sample size, high dimensionality, and strong correlations) would help confirm the robustness and broader clinical utility of these imputation methods. Because imputations introduce reconstructed rather than directly observed values, they can affect diagnostic thresholds, growth trend estimations, or predictive models used in surgical planning. While imputation improves research feasibility, its downstream impact on individual patient management underscores the need for cautious interpretation. The rarity of this condition limits subject availability, restricting generalizability of the findings and increasing statistical variability. Nevertheless, our evaluation demonstrates that RF imputation is a feasible approach for reconstructing incomplete craniofacial datasets. Future studies using larger dataset from multicentre cohorts should validate these methods to ensure they provide not only statistical accuracy, but also provide clinically reliable inputs for surgical planning and growth prediction. 

## Conclusion

This study evaluates the performance of different imputation methods on craniofacial morphometric analyses with data missing completely at random. Among the evaluated methods tested, MICE demonstrated lower imputation accuracy compared to others, whereas RF achieved the best balance between accuracy and variance preservation. The results suggest that RF is the most suitable option for handling missing data in small, high-dimensional and highly multicollinear datasets such as craniofacial measurements.

## Figures and Tables

**Fig. 7:**
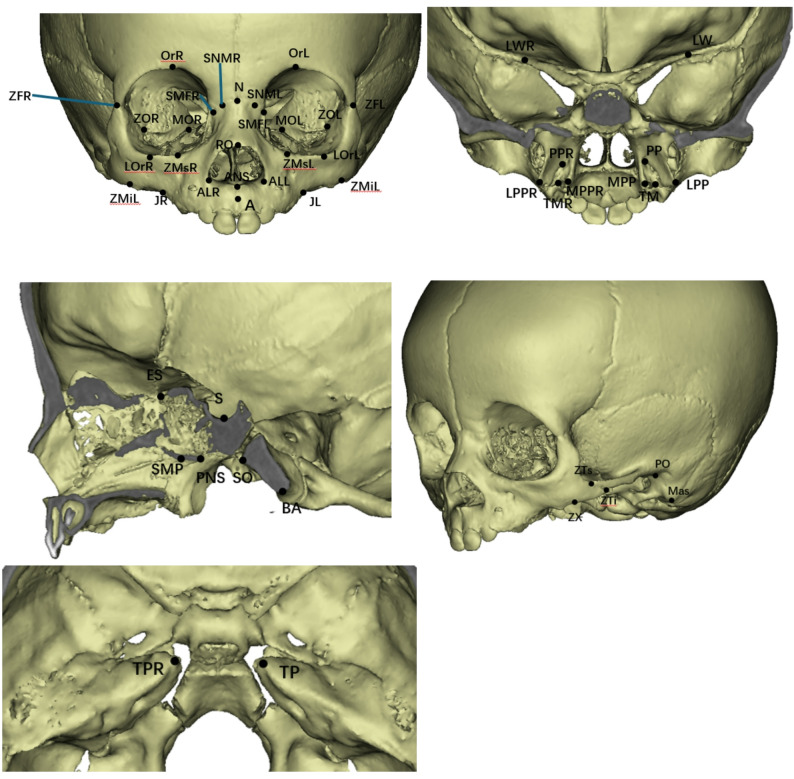
Location of each landmark in 3D skull structure

## Data Availability

The datasets and the R code used for this study are available here ([https://doi.org/10.22452/RD/4YR5BX](10.22452/RD/4YR5BX)).

## Appendix 1

**Table 5:** Definitions of the landmarks

ABBREVATION	DEFINITION
A	Point of maximum concavity in the midline of the alveolar process of the maxilla
AL(L/R)	The most lateral point on the piriform foramen in the lateral plane
ANS	It is the anterior tip of the sharp bony process of the maxilla in the middle of the lower margin of anterior nasal opening
BA	Most inferior and posterior point on the most anterior margin of the foramen magnum
ES	Most superior point of the suture between ethmoid and sphenoid at the midline
J(L/R)	The lateral point of middle maxilla
LOr(L/R)	The inferior point of the orbit
LPP(L/R)	The most inferior and posterior point of lateral sphenoid plate
LW(L/R)	The furthest point of the lesser wing of sphenoid
Mas(L/R)	The point that located posterior and inferior to the ear canal, lateral to the styloid process
MO(L/R)	The furthest point of maxilla in orbital floor
MPP(L/R)	The most inferior and posterior point of medial sphenoid plate
N	The most anterior point midway between the frontal and nasal bones on the fronto-nasal suture
Or(L/R)	The superior point of the orbital
PNS	Most posterior midpoint of the posterior nasal spine of the palatine bone.
Po(L/R)	The superior point of external auditory canal
PP(L/R)	The intersection of lateral and sphenoid plate
Ro	The anterior tip at the end of the suture of the nasal bones
S	Center of the hypophyseal fossa
SMF(L/R)	The innermost point of maxillafrontal suture
SMP	The inferior point of the maxilla palatine suture
SNM(L/R)	The superior point of the suture between the nasal bone and the maxilla
SO	Most anterior point on the midline of the occipital bone at the spheno-occipital synchondrosis
TM(L/R)	the most inferior and posterior point of maxilla
TP(L/R)	The intersection of the temporal and sphenoid body at the skull base
ZF(L/R)	The intersection of the medial points of the zygomatic frontal suture on both sides
ZMi(L/R)	The end point of the maxillary transition with the zygomatic bone, Point closed to alveolar process
ZMs(L/R)	Point at the zygomaticomaxillary suture on the anterior margin of the inferior orbital rim on each side
ZO(L/R)	The most inside point of zygoma in orbital side
ZTi(L/R)	The inferior point of the zygomaticotemporal suture
ZTs(L/R)	The superior point of the zygomaticotemporal suture
ZX(L/R)	Most inferior point of the inferior border of the zygomatic bone
